# Transcriptome Profiling Analysis Reveals Co-Regulation of Hormone Pathways in Foxtail Millet during *Sclerospora graminicola* Infection

**DOI:** 10.3390/ijms21041226

**Published:** 2020-02-12

**Authors:** Renjian Li, Yanqing Han, Qi Zhang, Guorong Chang, Yuanhuai Han, Xukai Li, Baojun Zhang

**Affiliations:** 1 College of Agriculture, Shanxi Agricultural University, Taigu 030801, Shanxi, China; renjianli1995@gmail.com (R.L.); xiaoqizhang1996@gmail.com (Q.Z.); changguorong1998@gmail.com (G.C.);; 2 College of Life Sciences, Shanxi Agricultural University, Taigu 030801, Shanxi, China

**Keywords:** *Sclerospora graminicola* (Sacc.) Schroeter, *Setaria italica*, gibberellin, endogenous hormones, transcriptome

## Abstract

*Sclerospora graminicola* (Sacc.) Schroeter is a biotrophic pathogen of foxtail millet (*Setaria italica*) and increasingly impacts crop production. We explored the main factors for symptoms such as dwarfing of diseased plants and the “hedgehog panicle” by determining panicle characteristics of varieties infected with *S. graminicola* and analyzing the endogenous hormone-related genes in leaves of Jingu 21. Results indicated that different varieties infected by *S. graminicola* exhibited various symptoms. Transcriptome analysis revealed that the ent-copalyl diphosphate synthetase (*CPS*) encoded by Seita.2G144900 and ent-kaurene synthase (*KS*) encoded by Seita.2G144400 were up-regulated 4.7-fold and 2.8-fold, respectively. Results showed that the biosynthesis of gibberellin might be increased, but the gibberellin signal transduction pathway might be blocked. The abscisic acid (ABA) 8′-hydroxylase encoded by Seita.6G181300 was continuously up-regulated by 4.2-fold, 2.7-fold, 14.3-fold, and 12.9-fold from TG1 to TG4 stage, respectively. Seita.2G144900 and Seita.2G144400 increased 79-fold and 51-fold, respectively, at the panicle development stage, promoting the formation of a “hedgehog panicle”. Jasmonic acid-related synthesis enzymes *LOX2s*, *AOS*, and *AOC* were up-regulated at the early stage of infection, indicating that jasmonic acid played an essential role in early response to *S. graminicola* infection. The expression of *YUC*-related genes of the auxin synthesis was lower than that of the control at TG3 and TG4 stages, but the amidase encoded by Seita.2G313400 was up-regulated by more than 30-fold, indicating that the main biosynthesis pathway of auxin had changed. The results suggest that there was co-regulation of the hormone pathways during the infection of foxtail millet by *S. graminicola*.

## 1. Introduction

Downy mildew disease of foxtail millet (*Setaria italica* (L.) P. Beauv) is caused by the oomycete *Sclerospora graminicola* (Sacc.) Schroeter, whose genome sequence was completed by Kobayashi et al. in 2017. The disease is prevalent in India, China, Japan, and Russia [1]. The incidence of downy mildew disease in China ranges between 20% and 30% but the infection rates of some susceptible varieties can exceed 70% in some years, and thus seriously affects foxtail millet yield and quality. Symptoms of this disease include rotten buds at the germination stage, downy mildew on the abaxial leaf surface known as “gray back”, decomposition of leaf mesophyll cells with only veins left as “white hairs”, and phyllody or witches’ broom as “hedgehog panicle”, as well as other morphological changes such as dwarfing, shortened internodes, and widened leaves. At present, there are few studies on the interaction between *S. graminicola* and foxtail millet, but some studies on the infection of pearl millet by *S. graminicola* have been reported. Lavanya et al. discovered that lipopolysaccharide can induce systemic resistance in pearl millet to *S. graminicola* [2]. To study the interaction between *S. graminicola* and pearl millet, Tiwari and Arya (1969) cultured the pathogen on host callus on medium [3]. It is shown that the transcripts and protein levels of allene oxide synthase (*AOS*) are significantly higher in resistant cultivars in comparison with those in susceptible cultivars, suggesting that *AOS* may play an important role in the response of pearl millet to *S. graminicola* [4].

Endogenous hormones not only regulate plant growth and development but are also involved in plant resistance to disease [5]. Salicylic acid (SA), abscisic acid (ABA), and jasmonic acid (JA) act as signal molecules to stimulate plant immune defense responses. Ethylene (ETH), auxin (IAA), gibberellin (GA), and cytokinin (CTK) are involved in plant abiotic stress resistance and also participate in the regulation of plant responses to disease-based biological stress [6,7,8]. When interacting with host plants, some pathogens can change normal plant growth and development processes by producing related plant hormones or by interfering with the balance of host plant endogenous hormones [9]. In mango exhibiting the witches’ broom malformation, plant GA and CTK contents are higher during the early period of pathogenesis and throughout the whole process [10]. Rice dwarf virus (RDV) coat protein P2 can interact with ent-kaurene oxidase (*KO*), a key enzyme in the rice GA biosynthetic pathway in RDV-infected rice to inhibit the synthesis of GA and the antitoxin phytoalexin, thereby boosting RDV levels and leading to dwarfing symptoms [11]. Jujube witches’ broom is caused by a phytoplasma. When the disease occurs, jujube floral organs are often transformed into leaves and internodes are shortened, due to triggering of floral organ degeneration and axillary bud production by the phytoplasma affecting IAA and CTK contents in the host [12]. Head smut in gramineous plants is caused by the sporadic parasitic pathogen *Sporisorium reilianum*. The disease symptoms appear in adult plants and mainly include blackened ears and malformed panicles. The pathogen is parasitic in vascular bundle tissues, and IAA secreted by the pathogen leads to the transformation of floral organs into vegetative organs [13,14].

Transcriptome sequencing is a high-throughput technology for studying expression at the genomic level, which has been used to identify the key genes involved in biological processes. It has also been applied extensively in the interaction between plants and pathogens. By using transcriptome sequencing technology, Albor et al. analyzed the early interaction between wheat and *Puccinia striiformis* and demonstrated that the pathogen may successfully colonize the host by inhibiting the expression of immune genes in wheat [15]. Du et al. identified a total of 655 MYC2-targeted JA-responsive genes in tomato infected with *Botrytis cinerea* using chromatin immunoprecipitation sequencing coupled with transcriptomic profiles [16]. By the combination of untargeted metabolomics and RNA sequencing, Jeon et al. revealed the role of the falcarindiol biosynthetic gene cluster in the resistance to fungal and bacterial pathogens in tomato leaves [17].

Although downy mildew has been widespread on foxtail millet in recent years in China, it is not clear if endogenous hormones are involved in the interaction between the pathogen and host. In this study, we therefore investigated the effect of downy mildew on plant height, panicle length, and the diameter and length of the panicle neck in foxtail millet varieties with different levels of resistance to the pathogen. According to our research, IAA, GA, JA, and ABA play important roles in the interaction between *S. graminicola* and foxtail millet. Transcriptome sequencing was used to analyze the expression profiles of genes related to endogenous hormone biosynthesis and signal transduction in leaves of foxtail millet infected with *S. graminicola*. The results of our experiments provide insights into the potential role of endogenous hormones in “hedgehog panicle” and dwarfing symptoms in foxtail millet.

## 2. Results

### 2.1. S. graminicola Caused Dwarfing and “Hedgehog Panicle” in Foxtail Millet

**Figure 1:**
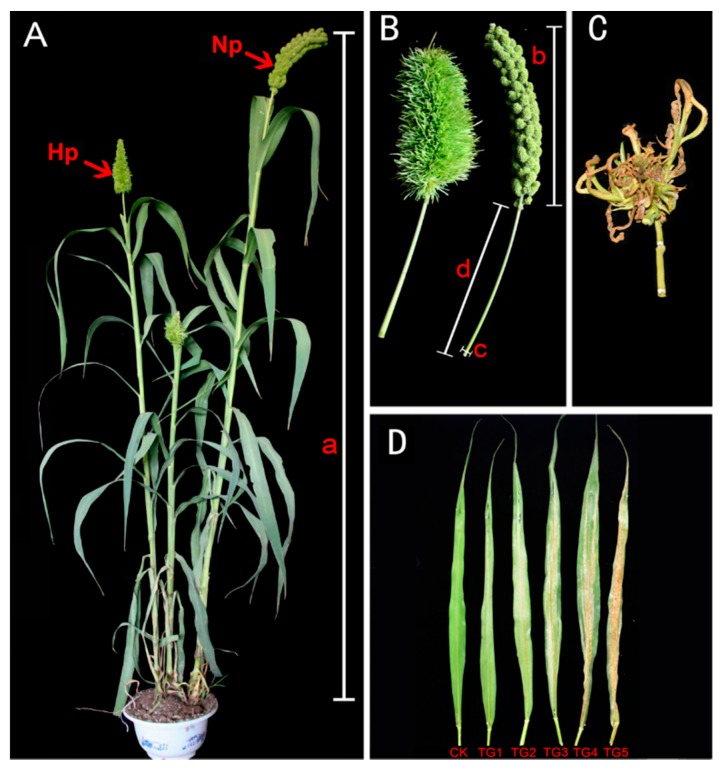
Downy mildew disease symptoms, “hedgehog panicle” and “gray back” in foxtail millet. (**A**) Healthy and downy mildew-infected foxtail millet plants, bearing normal panicle (Np) and “hedgehog panicle” (Hp), respectively. (**B**) Close-ups of “hedgehog head” and normal panicle, plant height (a), panicle length (b), panicle internode diameter (c), and length (d). (**C**) The panicle of witches’ broom. (**D**) Foxtail millet leaves showing the progression of “gray back” downy mildew symptoms. CK, uninfected healthy leaves; TG1–TG5, leaves at different stages of “gray back” development.

**Figure 2:**
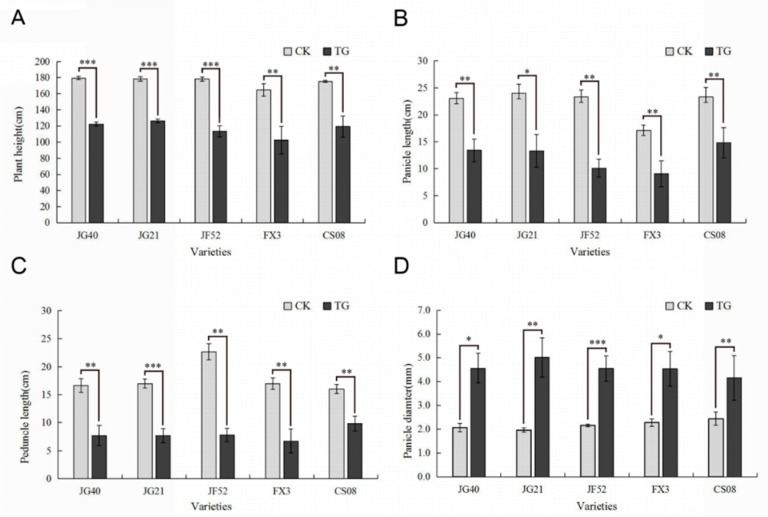
Phenotypes of downy mildew-resistant and -susceptible foxtail millet varieties. (**A**) Plant heights, (**B**) panicle diameters, (**C**) panicle lengths, (**D**) panicle internode lengths of resistant and susceptible foxtail millet cultivars. CK, control plants; TG, infected plants. Expression levels were analyzed using a T-test. Within each figure, asterisks above bars indicate statistical significance (*, *p* < 0.05; **, 0.01 < *p* < 0.05; ***, *p* < 0.01); Experiments were repeated at least three times with similar results.

Two typical manifestations of downy mildew disease in foxtail millet are “gray back” and “hedgehog panicle” (Figure 1A–D,). The extent of internode shortening and dwarfing in resistant and susceptible foxtail millet varieties was evaluated on plants infected by *S. graminicola*. The average height of infected Jinfen 52 plants was 113.43 cm, which was 64.90 cm shorter than that of healthy ones. Although Changsheng 08 and Fenxuan 3 are relatively resistant, their average heights were 102.27 cm and 119.13 cm when infected, with height reductions of 62.40 cm and 56.20 cm, respectively, in comparison with the controls (Figure 2A). After infection, the panicles of susceptible varieties were obviously shortened or exhibited a “hedgehog panicle” morphology, with a thickened panicle axis, an elongated panicle stalk, and an overall contracted panicle. Among the different varieties, the average lengths of panicles and their necks for infected Jinfen 52 plants were 10.10 cm and 7.80 cm, respectively, which were 13.23 cm and 14.87 cm shorter than those of uninfected plants (Figure 2B,C). Compared with uninfected plants, the panicle diameter in Jingu 21 increased by 3.04 cm on average (Figure 2D). These results showed that the degree of symptoms had no significant relationship with host resistance.

### 2.2. Identification of the Hormone-Associated Genes

**Figure 3:**
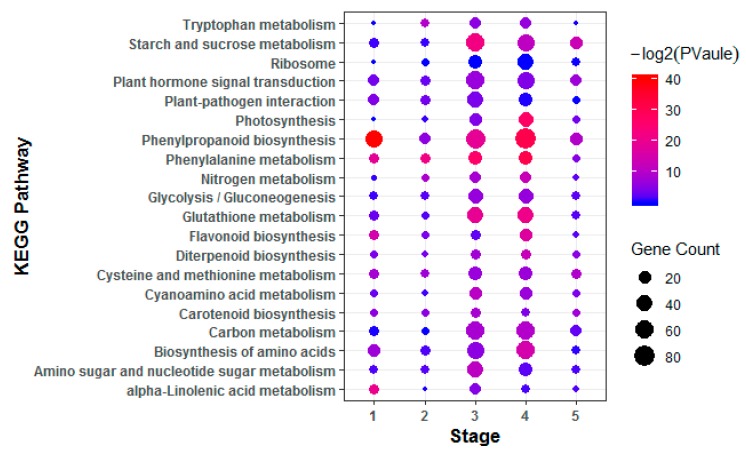
Enriched Kyoto Encyclopedia of Genes and Genomes (KEGG) pathways of the differentially expressed genes (DEGs). KEGG pathways with corrected *p*-value < 0.05 were considered significantly enriched.

**Figure 4:**
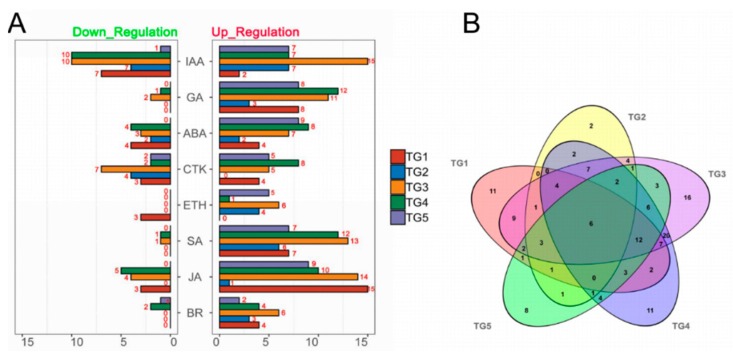
Statistics of hormone-associated genes at different stages of *S. graminicola* infection. (**A**) Numbers of hormone-associated DEGs at different stages (|Fold-change| ≥2). (**B**) Venn diagram showing the numbers of DEGs (|Fold-change| ≥2) at each stage with infection.

To investigate hormone-associated gene expression during *S. graminicola* infection, we collected infected leaves of Jingu 21 at the middle jointing, late jointing, booting, heading, and filling stages, and performed RNA-seq and data analysis. To further determine the involvement of hormone-associated metabolic pathways at the different stages we compared Kyoto Encyclopedia of Genes and Genomes (KEGG) enrichment results for the differentially expressed genes (DEGs) at each stage (Figure 3, Supplementary Table S2). In the KEGG enrichment results, the plant–pathogen interaction pathway was significantly enriched, and the number of enriched genes was largest at the TG3 and TG4 stages. Pathways related to plant hormone biosynthesis were also significantly enriched, and DEGs related to SA biosynthesis in hormones were significantly enriched with the largest numbers of genes at the TG1, TG3, and TG4 stages. DEGs related to auxin biosynthesis in tryptophan metabolism (map ko00380), the diterpenoid biosynthesis pathway (map ko00904) related to GA biosynthesis, the phenylalanine metabolism pathway (map ko00360) related to SA biosynthesis, the ETH biosynthesis pathway cysteine and methionine metabolism (map ko00270), and the JA biosynthesis pathway alpha-linolenic acid metabolism (map ko00592) were also enriched. The hormone signal transduction pathway was also significantly enriched at different stages. These results indicated that the regulation of hormones is very important in the process of *S. graminicola* infection. We statistically analyzed the genes associated with eight hormones involved in plant development at different stages (Figure 4A). In total, 261 hormone metabolism genes were up-regulated at the five stages, and 86 genes were down-regulated. The hormones with the most up-regulated genes were JA (49) followed by SA (45). The results showed that JA and SA played important roles in the response to *S. graminicola* infection. Among the significantly down-regulated hormone genes, auxin genes comprised the largest number (32), while SA genes were the least frequent (only 2 DEGs were significantly down-regulated). In the Venn diagram results (Figure. 4B), 16 DEGs were only expressed at the TG3 stage and 2 DEGs were only expressed at the TG2 stage. Additionally, 6 DEGs were involved in regulation in the TG5 stages, namely Seita.1G240600 (SA), Seita.9G351400 (GA), Seita.2G144400 (GA), Seita.6G181300 (ABA), Seita.4G224700 (BR), and Seita.5G154800 (ETH).

### 2.3. Hierarchical Clustering Analysis of the Expression Patterns of Hormone-associated Genes

**Figure 5:**
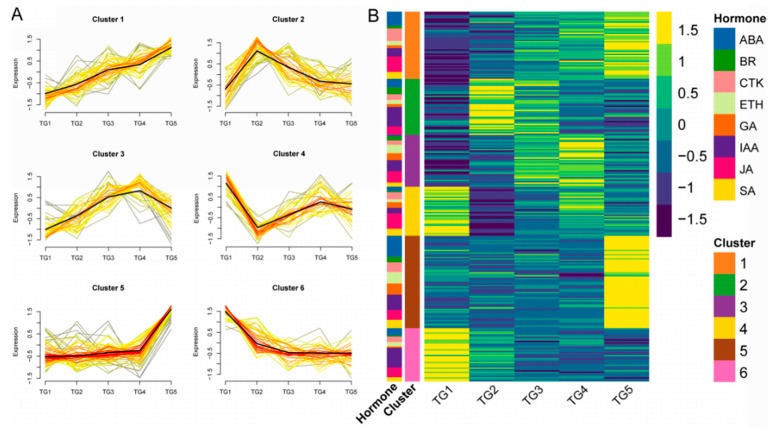
The expression patterns of hormone-associated genes by hierarchical clustering analysis. (**A**) Each square represents a pattern. Each line indicates a gene in this cluster.The color lines indicate the genes that better fit the overall expression trend in this cluster, as indicated by the black line, as they appear darker and closer to the black line. (**B**) Cluster expression heatmap of hormone-associated genes. The annotation on the left side of the heatmap shows the distribution of hormones in each cluster.

Hierarchical clustering analysis of the hormone-associated genes obtained was conducted (Figure 5A,B). Six types of expression patterns (clusters) were identified. There were 48 genes in cluster 1, with ABA and JA genes being the most common (10 and 11 genes, respectively). Cluster 2 contained 40 genes, mainly associated with IAA (14 genes). Cluster 3 contained 35 genes, with IAA and JA genes comprising the largest number (8 genes each). There were only 35 genes in cluster 4, with JA genes being the most abundant (11 genes). Cluster 5 and cluster 6 had 66 and 38 genes, respectively, among which IAA regulation genes represented the largest numbers, with 11 and 14 genes, respectively.

### 2.4. Identification of Hormone-associated Genes at Different Stages

**Figure 6:**
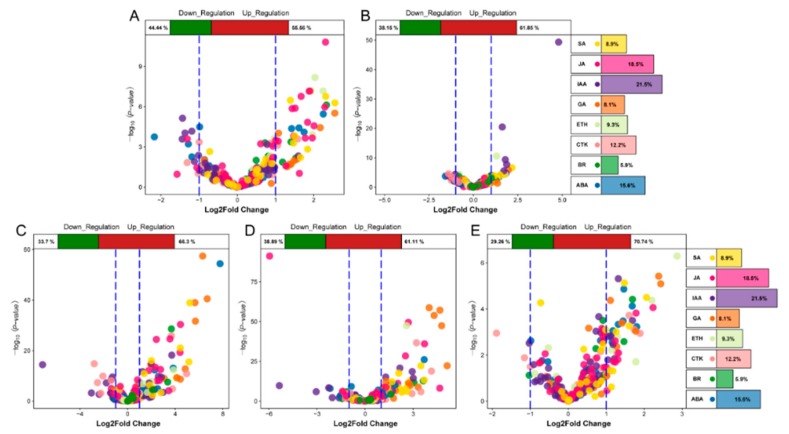
Volcanic plot of hormone-associated genes. The top of the volcano plot shows the proportion of up-regulated and down-regulated genes. The right side of the volcano plot shows the ratio of the number of hormone-associated genes. Different colors represent specific hormone types. (**A**,**B**) represent volcano plots of hormone-associated genes during jointing stages. (**C**,**D**,**E**) represent volcano plots of hormone-associated genes during panicle development.

We analyzed the differential expression of genes associated with eight hormones in the five stages and found that the DEGs at the TG1 stage were mainly JA, SA, ABA, auxin, and GA genes (Figure 6A,B). Among them, the number of JA-related genes was the highest, including 12 genes with a difference of more than 2-fold. At the TG3, TG4, and TG5 stages, the DEGs were mainly GA, SA, JA, IAA, and ABA genes (Figure 6C–E). The most significant difference was observed for xanthoxin dehydrogenase (*ABA2*) encoded by Seita.9G041800, which was up-regulated 220-fold at the TG3 stage. Most of the genes associated with GA were up-regulated. The expression of the ent-copalyl diphosphate synthase (*CPS*) encoded by Seita.9G351400, the ent-kaurene synthase (*KS*) encoded by Seita.2G144400, and the GA 3-beta-dioxygenase (*GA3oX*) encoded by Seita.5G125500 were increased by more than two times at each of the TG3, TG4 and TG5 stages.

### 2.5. S. graminicola Alters the Accumulation of Gene Transcripts in Hormone Biosynthesis Pathways in Foxtail Millet

**Figure 7:**
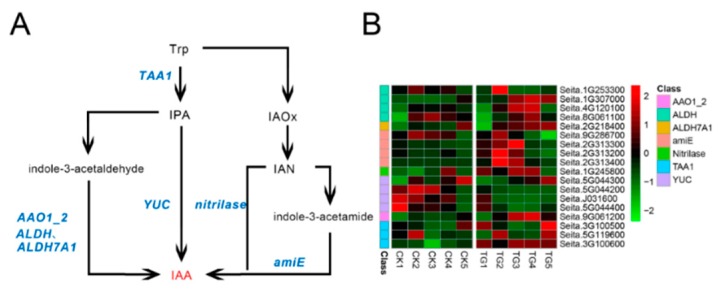
Auxin biosynthetic pathway and differentially expressed genes. (**A**) Auxin biosynthetic pathways. (**B**) Heatmap of differentially expressed genes related to this pathway. The FPKM (Fragments per kilobase of exon model per million mapped fragments) value of the gene was normalized by Pheatmap package in R language before the heatmap was drawn. In the color legend on the right side of the heatmap, the color labels of each gene correspond to the bar graph on the left side of the heatmap. The experiment was repeated at least three times and the results were similar. The heatmap data are the average of the repeated FPKM values.

To determine the effects of *S. graminicola* infection on endogenous hormone biosynthesis-related genes in foxtail millet, transcriptome sequencing was performed on leaves at different developmental stages. The indole-3-pyruvic acid (IPA) pathway is a key pathway for IAA biosynthesis. In infected plants, the expression of l-tryptophan-pyruvate aminotransferase (*TAA1*) generally increased at the TG3 and TG4 stages, whereas the expression levels of indole-3-pyruvate monooxygenase (*YUC*) genes, corresponding to Seita.5G044200, Seita.J031600, and Seita.5G044400, were lower than those in the control group and were significantly down-regulated at TG3 and TG4 (Figure 7, Supplementary Table S2). For example, the *YUC* gene Seita.J031600 was down-regulated 5.3-fold at TG3 and 8.3-fold at TG4 in comparison to the control. In the indole acetaldehyde oxime (IAOx) pathway, four genes encoding amidase (*amiE*), a key synthetase, were significantly up-regulated; in particular, the expression of Seita.2G313400 in infected foxtail millet was 34-fold higher than in the control at the TG2 stage and 30-fold higher at TG3. The transcript level of indole-3-acetaldehyde oxidase (*AAO1_2*) encoded by Seita.9G061200 was highest at the TG4 stage, while the expression level of acetaldehyde dehydrogenase was higher in the TG3 and TG4 stages.

**Figure 8:**
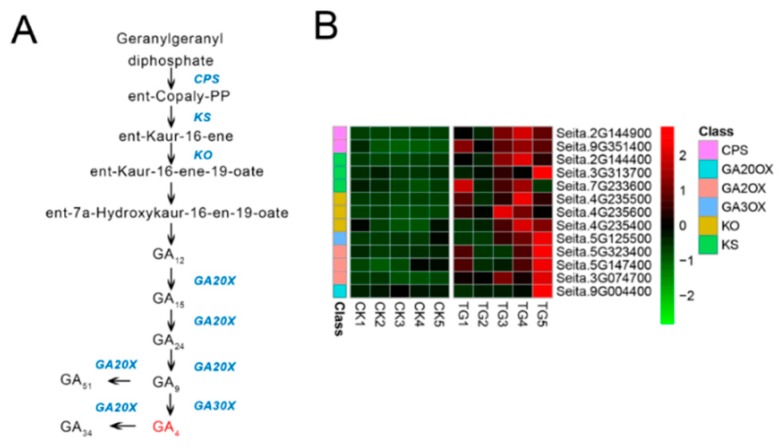
Gibberellin biosynthetic pathway and differentially expressed genes. (**A**) Gibberellin biosynthetic pathway. (**B**) Heatmap of differentially expressed genes related to this pathway. The FPKM values of the genes were normalized by Pheatmap package in R language before the heatmap was drawn. In the color legend on the right side of the heatmap, the color labels of each gene correspond to the bar graph on the left side of the heatmap. The experiment was repeated at least three times and the results were similar. The heatmap data are the average of the repeated FPKM values.

Six gene families comprising 13 genes were involved in GA biosynthesis (Figure 8). Compared with control plants, GA biosynthesis genes in infected plants showed significantly increased expression, including two genes annotated as ent-copalyl diphosphate synthetase (*CPS*) and three genes annotated as ent-kaurene synthase (*KS*). In particular, the *CPS* encoded by Seita.2G144900 and KS encoded by Seita.2G144400 were up-regulated 4.7- and 2.8-fold at the TG1 stage and up-regulated 79- and 51-fold at the TG3 stage, respectively. Two genes related to gibberellin 2-oxidase (*GA2oX*) were up-regulated 3- and 3.8-fold at TG1. Three genes related to ent-kaurene oxidase (*KO*) were up-regulated at the TG3 and TG4 stages, among which Seita.4G235500 and Seita.4G235600 were up-regulated 51.8- and 103-fold at the TG3 stage and 27.4- and 51.8-fold at the TG4 stage, respectively. Genes encoding key GA biosynthetic enzymes were significantly up-regulated in infected plants at the TG3 stage, which corresponds to the critical stage of panicle formation, the booting stage.

**Figure 9:**
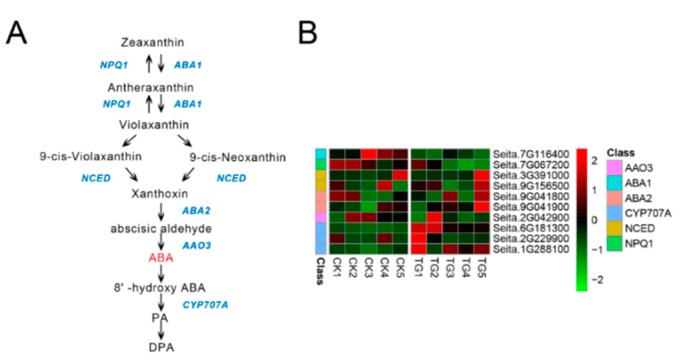
Abscisic acid biosynthetic pathway and differentially expressed genes. (**A**) Abscisic acid biosynthetic pathway. (**B**) Heatmap of differentially expressed genes related to this pathway. The FPKM values of the genes were normalized by Pheatmap package in R language before the heatmap was drawn. In the color legend on the right side of the heatmap, the color labels of each gene correspond to the bar graph on the left side of the heatmap. The experiment was repeated at least three times and the results were similar. The heatmap data are the average of the repeated FPKM values.

Seven genes involved in ABA biosynthesis and three genes involved in ABA metabolic reactions showed gradually decreased expression from TG1 to TG4, reaching a minimum at the TG4 stage before their transcript levels increased at TG5 (Figure 9). The key enzyme of ABA biosynthesis, 9-cis-epoxycarotenoid dioxygenase (*NCED*), was expressed at the TG1, TG2, and TG5 stages, with the expression of the *NCED* encoded by Seita.9G156500 increasing 3.2-fold at TG5. The ABA 8-hydroxylase (*CYP707A*) Seita.6G181300 was highly expressed at the TG1 and TG2 stages in the infected plants, being up-regulated 4.7-fold and 2.7-fold, respectively, with low or no expression at the other stages.

**Figure 10:**
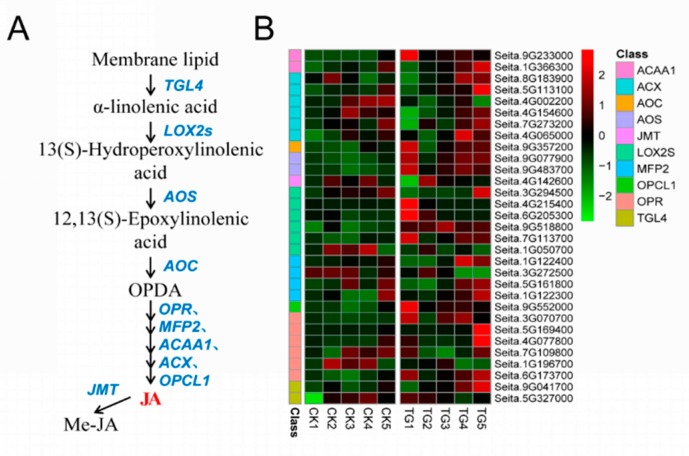
Jasmonic acid biosynthetic pathway and differentially expressed genes. (**A**) Jasmonic acid biosynthetic pathway. (**B**) Heatmap of differentially expressed genes related to this pathway. The FPKM values of the genes were normalized by Pheatmap package in R language before the heatmap was drawn. In the color legend on the right side of the heatmap, the color labels of each gene correspond to the bar graph on the left side of the heatmap. The experiment was repeated at least three times and the results were similar. The heatmap data are the average of the repeated FPKM values.

Ten gene families comprising 31 genes were involved in JA biosynthesis. The key JA synthetases *LOX2*, *AOS*, *OPR*, and *OPCL1* were all significantly up-regulated during pathogen infection (Figure 10). Among them, the expression of lipoxygenase encoded by Seita.6G205300, Seita.9G518800, and Seita.7G113700 was more than 2 times different at the TG1 and TG3 stages. The *AOS* genes encoded by Seita.9G077900 and Seita.9G483700 were highly expressed at TG1, TG3, TG4, and TG5; in particular, Seita.9G483700 was up-regulated 22 times at TG3.

### 2.6. S. graminicola Alters Transcripts of Genes in the Hormone Signal Transduction Pathway in Foxtail Millet and Verification by Quantitative Real-Time PCR

**Figure 11:**
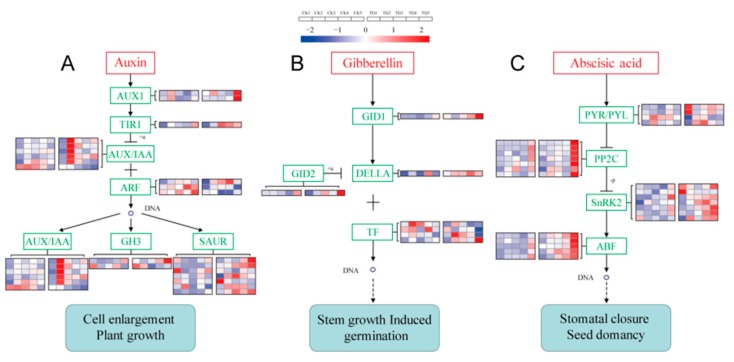
Signal transduction pathways and expressions of related genes. (**A**) Auxin signal transduction. (**B**) Gibberellin signal transduction. (**C**) Abscisic acid signal transduction. The FPKM values of the genes were normalized by Pheatmap package in R language before the heatmap was drawn. In the color legend on the right side of the heatmap, the color labels of each gene correspond to the bar graph on the left side of the heatmap. The experiment was repeated at least three times and the results were similar. The heatmap data are the average of the repeated FPKM values. In the heatmap, five on the left side of the heatmap box are CK1-5, and on the right side of the heatmap box are TG1-5, respectively.

Genes related to the IAA signal transduction pathway such as IAA/indole-3-acetic acid (*AUX/IAA*), Gretchen Hagen3 (*GH3*), and small-IAA-up-RNA (*SAUR*) genes showed significant responses in *S. graminicola*-infected plants (Figure 11A, Supplementary Table S2). The transport inhibitor response 1 (*TIR1*) gene Seita.3G037200 was highly expressed at the TG3 and TG4 stages. Seita.3G080900, annotated as *AUX/IAA*, was expressed at the TG2, TG3, and TG5 stages. The IAA response factor gene (*ARF*) Seita.1G195500 was expressed at the TG2, TG3, and TG4 stages and was significantly down-regulated at TG4. The accumulation of transcripts for the GA signal transduction pathway DELLA protein encoded by Seita.9G123000 showed almost no change compared with the control group, with fold changes less than 2. Phytochrome-interacting factor 3 (*TF3*) and phytochrome-interacting factor 4 (*TF4*) genes, which are involved in GA signal transduction, were also affected. The TF3 encoded by Seita.5G034000 was down-regulated 2.2-fold at the TG3 stage (Figure 11B, Supplementary Table S2). Compared with the control group, the ABA receptor *PYR/PYL* family genes Seita.9G311900 and Seita.9G437300 were highly expressed at the TG1 stage, being up-regulated 2.7- and 3.3-fold, respectively. The gene encoding *SnRK2* was highly expressed at TG1 and significantly up-regulated more than 2-fold at TG3 and TG4. The ABA responsive element binding factor gene (*ABF*), which was only very weakly expressed at the TG1 stage, was highly expressed at the TG3, TG4, and TG5 stages, being up-regulated 3.7- and 2.2-fold at TG4 and TG5, respectively (Figure 11C, Supplementary Table S2).

To verify the reliability of the transcriptome data, six genes involved in hormone biosynthesis and signal transduction pathways were selected for qRT-PCR verification. A comparison of the log2-transformed transcriptome sequencing data and qRT-PCR data for these genes (Seita.1G190000, Seita.1G195500, Seita.3G246300, Seita.4G120100, Seita.9G061200, Seita.9G041900) indicated that the expression trends determined by the two techniques were basically the same (R2 = 0.84; Supplementary Figure S1), demonstrating the reliability of the RNA-seq results.

## 3. Discussion

**Figure 12:**
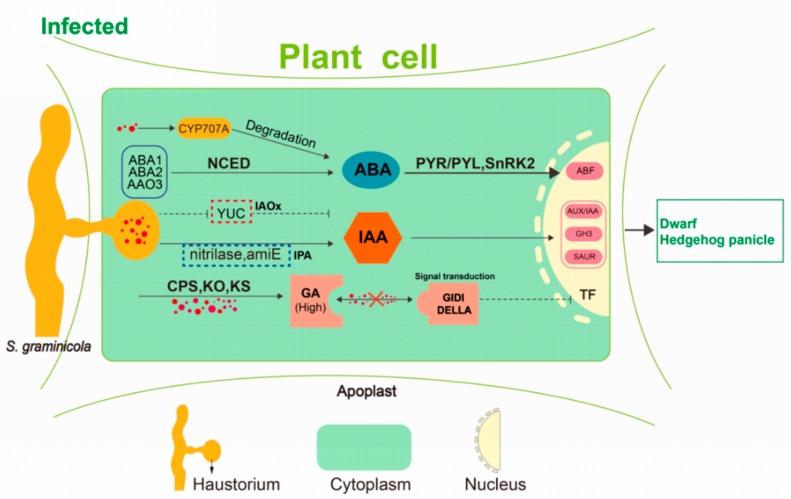
A hypothetical model of dwarfing and “hedgehog panicle” formation. *S. graminicola* colonization inhibits the expression of *YUC* enzyme and thus in the IPA pathway of auxin biosynthesis but activates the IAOx pathway regulated by *amiE* and nitrilase. These changes may be related to the disease-resistant response of foxtail millet. *S. graminicola* promotes *CPS*, *KO*, *KS,* and increases gibberellin content. Blocking of the gibberellin signal transduction also occurs, resulting in inhibition of gibberellin action. Abscisic acid accumulated to a relatively high level at the early developmental stage, and the *S. graminicola* then promoted the expression of abscisic acid degrading enzyme *CYP707A*, leading to the decrease of abscisic acid content.

Dynamic changes in plant endogenous hormones play important roles in plant-pathogen interactions [18]. The effect of *S. graminicola* infection on endogenous hormones in foxtail millet was the main cause of dwarfing and “hedgehog panicle” formation (Figure 12). The expression profiles of IAA biosynthesis-related genes revealed that there was a switch from the IPA pathway to the IAOx pathway of IAA synthesis during the development of the disease. Analysis of genes related to the IAA signal transduction pathway showed that the *TIR1* gene was highly expressed at the TG3 and TG4 stages. *TIR1* can cause *AUX/IAA* ubiquitination, relieving the inhibition of *AUX/IAA* on *ARF* activity and hence initiating the transcription of IAA-induced genes [19]. The expression level of the *ARF* gene (Seita.1G195500) was consistent with that of *TIR1*, and these two genes function together to activate the expression of *SAUR*-and *GH3*-related downstream genes. *SCT1b* is an important factor in the degradation of *AUX/IAA* by SCF (*TIR1*), and the *sgt1b* mutant of *Arabidopsis thaliana* can inhibit *rpp5*-mediated resistance [20]. Overexpression of GH3-8 in rice can enhance resistance to *Xanthomonas oryzae* pv. oryzicola [21], with an increase of IAA content that is probably a manifestation of host resistance.

According to our transcriptome analysis, the key synthetases *KS* and *KO* were significantly up-regulated at the TG1, TG2, and TG3 stages. In the GA signal transduction pathway, the phytochrome-interacting factor *TF3* was significantly down-regulated, which indicates that the GA signal transduction pathway might be blocked. DELLA proteins are usually located in the nucleus, where they can block GA signal transduction by inhibiting the transcription of TFs [22]. When the GA content increases, the N-terminal segment of the GA receptor GIDI interacts with the GA -lactolide ring, which causes a change in the conformation of the N-terminus. This complex binds with the DELLA protein to form a trimer, which relieves the inhibitory effect of DELLA on plant growth [23,24]. In our study, GA was abundant in foxtail millet after infection with *S. graminicola*, signal transduction pathways were suppressed, and the expression of the phytochrome-interacting factor *TF3* was significantly reduced. Chahtane et al. (2018) [25] reported that l-2-amino-4-methoxy-trans-3-butenoic acid secreted by *Pseudomonas aeruginosa* directly acts on DELLA proteins of the GA signal transduction pathway to inhibit seed germination in *Arabidopsis thaliana*, regardless of GA content. Our results suggest that *S. graminicola* infection might block the GA signal transduction. GA synthesized and accumulated in the infected host is therefore not sensed by the GA receptor; consequently, GA has no physiological effect, leading to dwarfing.

In the ABA biosynthesis pathway, we found that the ABA 8′-hydroxylase (*CYP707A*) encoded by Seita.6G181300 and Seita.1G288100 was up-regulated 4.7-fold and 3.1-fold, respectively. The reason for this may be that the ABA content in the host was too high, causing negative feedback regulation of *CYP707A* to promote ABA degradation and metabolism. Excessive ABA content in the host may also be an important factor related to dwarfing. The increased ABA content could promote the binding of the ABA receptor *PYR/PYL* with protein phosphatase 2C (PP2C) and inhibit its phosphatase activity, and subsequently remove its inhibition on serine/threonine-protein kinase SRK2 (*SnRK2*). *TaSnRK2.3*-1B was reported to negatively regulate plant height in wheat [26]. ABA 8′-hydroxylase may play a role in activating IAA and GA biosynthesis during *S. graminicola* infection.

To produce the “hedgehog panicle” symptom, floral organs are transformed into leaves (phyllody or witches’ broom). The plant hormones measured here are generally mobile in the plant. Whether hormone changes are the key factor in this aberrant panicle development, however, it is not certain: but it is clear that it is caused by genetic changes induced by *S. graminicola*. An imbalance between CTK and IAA is known to cause Jujube witches’ broom triggered by phytoplasmal infection, and it seems that in infected foxtail millet plants the development of panicles can either be partially or completely disrupted during disease development (Figure 1B,C). and it appears that infection leads to the abolition of normal floral development. There are genetic mutations that affect related processes. For example, mutation of *LHS1* in rice results in elongated inner and outer lemmas and abnormal paleas and lemma-like lodicules [27]. The roles and mechanisms of floral initiation and development genes in the process of “hedgehog panicle” formation, however, need further investigation.

## 4. Materials and Methods

### 4.1. Foxtail Millet Varieties, Inoculation, and Planting

The foxtail millet varieties (Jingu 21, Jingu 40, Jinfen 52, Fenxuan 3, and Changsheng 08) were provided by the Institute of Agricultural Bioengineering, Shanxi Agricultural University, Taigu, China. According to infection incidence statistics over 3 years (Supplementary Table S3), Jinfen 52, Fenxuan 3, and Changsheng 08 were disease-resistant varieties, while Jingu 21 and Jingu 40 were susceptible.

Oospores of the pathogen were isolated from infected foxtail millet leaves in late autumn in the experimental station at Shanxi Agricultural University. Leaves with brown lesions were collected, dried naturally in a cool place, gently crushed, and sifted to obtain oospores, which were stored at 4 °C for later use.

The collected oospores were mixed with seeds at a ratio of 1:5 by weight and sown in the field on May 8 2017. The plant spacing was 20 cm × 40 cm, and the plot area was 15 m^2^. Four replicates were performed, with seeds without oospores used as controls. During the grain-filling period in late September, plant height, panicle length, the diameter and length of the panicle neck, and other characteristic symptoms in infected and healthy plants of each variety were recorded.

Infected leaves of Jingu 21 were collected at the middle jointing, late jointing, booting, heading, and filling stages, dipped immediately in liquid nitrogen and then stored at −80 °C. Leaves of healthy plants were collected at the same stages for use as controls. Three replicates were obtained at each stage.

### 4.2. RNA Extraction, Library Construction, and Sequencing

Total RNA was extracted from 500 mg infected and healthy leaves of five developmental stages by the Trizol method [28]. Determination of RNA degradation by 1% agarose gel, and the RNA integrity and concentration, were checked using an Agilent 2100 Bioanalyzer (Agilent Technologies, Inc., Santa Clara, CA, USA). Poly-T oligo-attached-magnetic beads were used to purify mRNA from total RNA and the enriched mRNA was fragmented into approximately 200 nt RNA inserts, which were used to synthesize the first-strand cDNA and the second cDNA. End-repair/dA-tail and adaptor ligation were performed on the double-stranded cDNA. The cDNA library was constructed following the manufacturer’s instructions of NEBNext Ultra RNA Library Prep Kit for Illumina (NEB, E7530, Ipswich, USA) and NEBNext Multiplex Oligos for Illumina (NEB, E7500, Ipswich, USA). The library fragments were purified with AMPure XP system (Beckman Coulter, Beverly, Indianapolis, USA). Finally, the constructed cDNA libraries of the leaves were sequenced on a flow cell using an Illumina HiSeq™ sequencing platform.

### 4.3. Bioinformatic Analysis of Transcriptome Data

The generated sequencing data were evaluated using the Trimmomatic software [29], and the filtered clean data were compared against the foxtail millet genome (*Setaria italica* v2.2: https://phytozome.jgi.doe.gov/pz/portal.html#!info?alias = Org_Sitalica) using the Hisat2 software [30]. Expression level quantification was performed in StringTie [31]. DESeq2 was used to assess differential expression between sample groups [32]. The differentially expressed genes (DEGs) were identified by applying a log2FC (fold change) for up-regulated and down-regulated genes, respectively, with *p*-value < 0.05. DEGs were functionally annotated using the KEGG database (https://www.kegg.jp/) [33]. Clustering was performed using Mfuzz package [34]. The Pheatmap R package was used to generate expression heatmaps [35].

### 4.4. Quantitative Real-Time PCR Analysis

Transcripts of six genes related to the synthesis and signal transduction pathways of IAA, GA, ABA, and other hormones were used for qRT-PCR verification. Extracted RNA was purified and reverse transcribed into cDNA using an M-MuLV First Strand cDNA Synthesis kit (Sangon Biotech, Shanghai, China). The primers used are listed in Supplementary Table S1. A 2×SG Fast qPCR Master Mix kit (Sangon Biotech, Shanghai, China) was used for the quantitative fluorescence assay. The qRT-PCR amplifications were performed in 20 μL reaction volumes consisting of 10 μL of 2×SG Fast qPCR Master Mix, 0.4 μL of each primer, 500 ng cDNA template, 2 μL DNA buffer, and PCR-grade water. The PCR setup was: initial denaturation at 95 °C for 3 min, followed by 40 cycles of denaturation at 95 °C for 3 s and annealing/extension at 60 °C for 30 s. The actin gene was used as an internal control, and relative gene expression was calculated by the 2CT method [36]. The experiments were repeated at least three times.

## 5. Conclusions

In this study, transcriptome sequencing was performed at different stages of *S. graminicola* infection, and the characteristic genes in the biosynthetic pathways and signal transduction pathways of different hormones were analyzed by transcriptome sequencing. It was found that *S. graminicola* may affect the growth and development of the host by inhibiting the GA signal transduction, thus leading to dwarfing symptoms. At the same time, *S. graminicola* infection affected the auxin biosynthesis pathway in the host. These results provide an experimental basis for the role of hormones in the pathogenesis of *S. graminicola*, and the mechanism whereby infection alters development, and also greatly inform further hybrid breeding efforts to control downy mildew in foxtail millet.

## Supplementary Materials

Supplementary materials can be found at https://www.mdpi.com/1422-0067/21/4/1226/s1.

## Abbreviations

*S.graminicola*	Sclerospora graminicola (Sacc.) Schroeter
GA	Gibberellin
IAA	Auxin
ABA	Abscisic acid
CTK	Cytokinin
ETH	Ethylene
SA	Salicylic acid
JA	Jasmonic acid
BR	Brassinosteroids
IPA	Indole-3-pyruvic acid
IAOx	Indole acetaldehyde oxime
TAA1	L-tryptophan---pyruvate aminotransferase
YUC	Indole-3-pyruvate monooxygenase
AAO1_2	Indole-3-acetaldehyde oxidase
ALDH	Acetaldehyde dehydrogenase
CPS	Ent-copalyl diphosphate synthetase
KS	Ent-kaurene synthase
KO	Ent-kaurene oxidase
NCED	9-cis-epoxycarotenoid dioxygenase
CYP707A	ABA 8-hydroxylase
GH3	Gretchen hagen3
AUX/IAA	IAA/indole-3-acetic acid
SAUR	Small IAA up RNA
TIR1	Transport inhibitor response 1
TF3	Phytochrome-interacting factor 3
TF4	Phytochrome-interacting factor 4
PYR/PYL	ABA receptor
PP2C	Protein phosphatase 2C
ABF	ABA responsive element binding factor
GID1	Gibberellin receptor 1
KEGG	Kyoto Encyclopedia of Genes and Genomes
