# Supplementary figures and images for: Transcriptome Profiling Analysis Reveals Co-Regulation of Hormone Pathways in Foxtail Millet during Sclerospora graminicola Infection
Source: Int J Mol Sci. 2020 Feb 12;21(4):1226. doi: 10.3390/ijms21041226 (PMC7072888; doi:10.3390/ijms21041226)

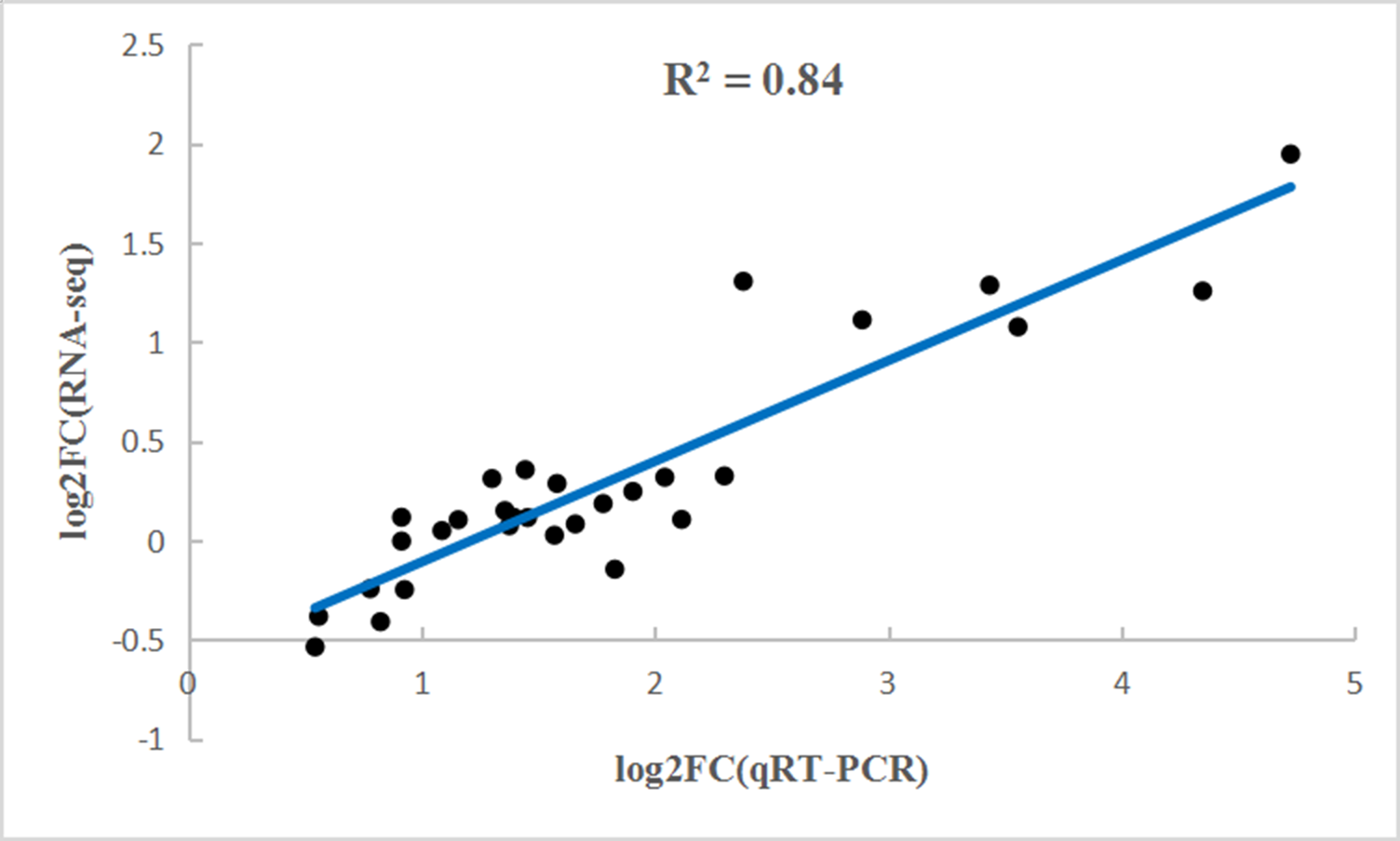

Supplement: Supplementary file 1 [file ijms-21-01226-s001.zip › ijms-659459 supplementary/Supplementary Figure S1.tif]
